# Pre-shot strategy selection and scoring performance in golf

**DOI:** 10.3389/fpsyg.2026.1838440

**Published:** 2026-06-22

**Authors:** Guowen Ai, Taihe Liang, Xianfei Wang, Zhenbang Zhou, Huanxin Tian, Shiao Zhao, Zhide Liang, Senyao Du, Ziheng Ning

**Affiliations:** 1 Institute of Physical Education, Hainan Normal University, Haikou, Hainan, China; 2 Faculty of Health Sciences and Sports, Macao Polytechnic University, Macao, China; 3 Hunan University, Changsha, China

**Keywords:** Bayesian analysis, decision making, ecological dynamics, golf, multilevel model

## Abstract

**Introduction:**

Pre-shot strategy selection in golf occurs under relatively low external time pressure, allowing players to become attuned to the affordances of the hole before executing the tee shot. The association between such pre-shot affordance engagement and scoring outcomes has, however, received limited empirical attention. Drawing on ecological dynamics, we conceptualised aggressive versus conservative tee-shot decisions on dogleg holes as differing forms of engagement with the affordance landscape, and examined how scoring outcomes were associated with this engagement both within and between players.

**Methods:**

Eighty-one male university (amateur) golfers (mean handicap = 25.99, SD = 9.50) played three dogleg holes on a single regulation course, choosing between an aggressive line that cuts across the corner and a conservative line that follows the fairway. Each player declared their intended strategy verbally before each tee shot; total strokes were recorded per hole and converted to score-to-par. A Bayesian multilevel model with within-between decomposition was applied to 243 observations to separate stable between-player strategic tendencies from situational within-player decision variation.

**Results:**

Within-player deviations toward aggressive decisions were credibly associated with higher scores relative to par [Estimate = 0.51, 95% CrI (0.11, 0.90), *p* (*β* > 0) = 0.993], amounting to approximately half a stroke per hole. The between-player effect of stable strategic tendency was inconclusive [Estimate = 0.26, 95% CrI (−0.29, 0.80)]. A random slope model provided exploratory evidence that the decision-performance association varied across individuals, with a positive intercept-slope correlation (*r* = 0.70) suggesting that players with weaker baseline performance showed larger within-player decision-performance associations; this finding was sensitive to prior specification and should be regarded as preliminary. The within-player association was robust to alternative regression-coefficient priors and to a cumulative ordinal model.

**Discussion:**

Within the present three-hole single-course design, situational within-player deviations were more informative than coarse player-type classifications. These observational findings support the application of within-between decomposition in sport decision-making research.

## 1 Introduction

### 1.1 Pre-shot strategy selection on dogleg holes

Sport decision-making occurs across different timescales and task constraints. Much empirical work has examined rapid decisions made under acute time pressure, including anticipatory cue use in interceptive actions (Abernethy et al., 2001), passing choices in team sports (Travassos et al., 2013a,b), and research on judgment and decision-making processes in sport more broadly (Johnson, 2025; Raab et al., 2019; Seidel-Marzi et al., 2024; Travassos et al., 2013b). Golf tee-shot strategy selection on dogleg holes represents a slower pre-shot context that has received comparatively less systematic study (Marasso et al., 2014): before executing the tee shot, players can visually explore the hole, become attuned to the available affordances, and orient their intended action toward the task goal without the acute temporal constraints typical of fast-paced interceptive tasks (Ozbeklik and Smith, 2017; Pope and Schweitzer, 2011). The association between these pre-shot engagements with the affordance landscape and actual scoring outcomes has not been systematically examined in the golf performance literature.

**Figure 1:**
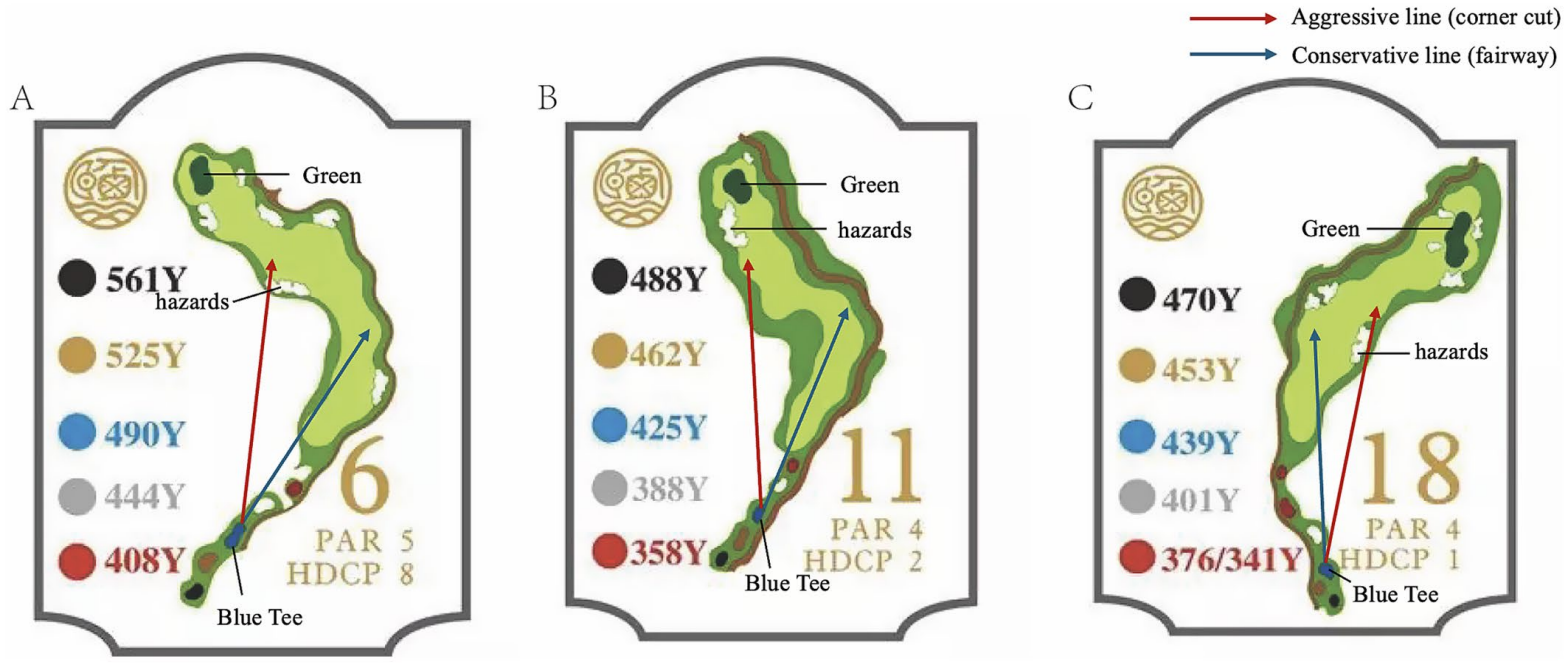
The three dogleg holes used in the present study, with the two pre-shot tee-shot strategies overlaid. Panel **(A)** Hole 6 (par 5, hole handicap 8, left dogleg); Panel **(B)** Hole 11 (par 4, hole handicap 2, left dogleg); Panel **(C)** Hole 18 (par 4, hole handicap 1, right dogleg). Blue Tee marks the tee box used by participants; Green marks the putting green; Hazards indicate fairway-side bunkers. The red arrow marks the aggressive line, which cuts across the dogleg corner to shorten the route to the green: the landing zone finishes closer to the green, but the tee shot is more likely to go out-of-bounds or to land in a bunker. The blue arrow marks the conservative line, which follows the fairway around the corner; landing zones along this line lie predominantly on the fairway and are therefore the safer choice, although the player may need an additional stroke to make up the distance that the aggressive line would otherwise have gained. Adapted, with permission, from the official course yardage chart of Hainan Guyantian Golf Club.

Before stating the hypotheses, we briefly clarify the golf-specific terminology used throughout the paper. A tee shot is the first stroke played on each hole, taken from a designated teeing area. A dogleg hole is a hole whose fairway bends between the tee and the green; on such holes, players choose between an aggressive line that attempts to carry over the corner of the dogleg (shortening the route to the green at the expense of a narrower margin for error) and a conservative line that follows the bend of the fairway (sacrificing distance for landing safety). A hole’s par is the standard number of strokes an expert player is expected to take on a given hole; score-to-par on a single hole is calculated as strokes taken minus par, so that positive values denote scores above par (poorer outcomes). Figure 1 illustrates the aggressive and conservative lines on the three dogleg holes used in the present study.

Beyond the structural description given above, what makes dogleg holes theoretically informative is that they require a strategic choice with a real performance trade-off (Smith, 2019). An aggressive line may yield a shorter approach to the green, but the demand for precision is higher and the penalty for deviation is greater. A conservative line reduces immediate risk at the expense of a longer approach. Existing discussions of golf strategy often assume that individual players possess relatively stable strategic dispositions, characterised as aggressive or conservative, that influence performance across situations (Iskra et al., 2025; McFall and Rotthoff, 2020). This player-type assumption has seldom been tested in designs capable of separating the contribution of stable tendencies from that of situation-specific choices.

### 1.2 Ecological dynamics, affordances, and skill–environment fit

Ecological dynamics offers an alternative theoretical account. Within this framework, behaviour in sport is understood as emerging from the continuous interaction between a performer and environmental constraints, rather than from stable internal dispositions alone (Araújo et al., 2006, 2019; Davids et al., 2013). Central to the framework is the concept of affordances: action possibilities that are relational properties defined by the fit between an individual’s capabilities and the structure of the performance environment (Gibson, 1979). The landscape of affordances on a dogleg hole is shared among all players, in that the corner invites cutting and the fairway invites safe positioning regardless of who stands on the tee. However, the field of relevant affordances, meaning the subset of possibilities that actually solicits a given player to act in a given situation (Kiverstein et al., 2021), is shaped by the relation between the individual’s skills, intentions and the task constraints. In the present task, this intention is defined by the golfer’s goal of advancing the ball toward the green under the scoring and rule constraints of golf, while selecting a tee-shot route that can be reliably realised with their current action capabilities. For a particular player, the aggressive route may be a realisable affordance or a deceptive one, depending on the fit between the execution demands of that route and the player’s actual capabilities. Skilled engagement with affordances requires situated normativity, the capacity to distinguish adequate from inadequate actions in a specific setting. Situated normativity develops not only through practice and experience but also in relation to the sociocultural constraints of the sport, in particular its rules, which enable some actions and penalise others, thereby contributing to a particular form of life (Gibson, 1979; Rietveld and Kiverstein, 2014). From this perspective, the within-player association between aggressive engagement and score-to-par is not uniform across players: those whose situated normativity is less developed may perceive aggressive routes as viable when they are not, resulting in wider affordance-capability mismatches and, on average, higher score-to-par.

Previous ecological dynamics studies have examined how performers become attuned to affordances by operationalising action possibilities through task constraints, environmental information, and performer capabilities; recent work has shown how experience shapes affordance-based control and perception in tasks such as braking in cycling and aperture crossing (Araújo et al., 2025; Gotardi et al., 2024; Vauclin et al., 2024). Comparatively less work, however, has examined how pre-shot affordance engagement in a real sport setting can be linked quantitatively to performance outcomes while separating stable between-player tendencies from situational within-player variation. The present study addresses this analytical gap by combining pre-shot verbal reports in an on-course golf task with a Bayesian multilevel within-between decomposition. We acknowledge that affordance perception and capability-affordance fit were not directly measured; the ecological dynamics framing is therefore offered as an account consistent with the data rather than a mechanism tested by the present design.

### 1.3 Previous golf decision-making research and the analytical gap

A critical methodological issue in sport decision-making research that draws on multilevel field data is that many studies do not distinguish between stable between-player differences in strategy preference and situational within-player variation in decision choice (Curran and Bauer, 2011; Rohrer and Murayama, 2023). When these two sources of variation are conflated, it is not possible to determine whether observed performance differences arise because certain players are dispositionally more aggressive, or because players choose different strategies in response to specific environmental contexts. The within-between modelling framework addresses this problem by decomposing each decision into a stable between-player component and a situational within-player component, allowing the effects of stable preferences and context-specific choices to be estimated independently.

Existing golf decision-making research, much of it situated in golf analytics and sports economics, has primarily examined professional samples and questions of risk preference, loss aversion, and aggregate scoring efficiency (Broadie, 2012, 2014; Broadie and Hurley, 2017; Ozbeklik and Smith, 2017; Pope and Schweitzer, 2011; McFall and Rotthoff, 2020). The present study differs from this body of work in two respects: it uses an amateur university sample with substantial variability in playing ability, and it pairs pre-shot verbal reports with a within-between decomposition to separate stable strategic tendencies from situational decision deviations on dogleg holes.

Research on action observation and predictive motor simulation has provided important insights into rapid anticipation in time-pressured sport tasks (Aglioti et al., 2008; Paolini et al., 2023, 2025; Rizzolatti and Craighero, 2004). The present study is adjacent to that literature but is not grounded in a motor-simulation account; instead, it examines a slower pre-shot golf context from an ecological dynamics perspective, focusing on how declared tee-shot engagement with dogleg affordances is associated with scoring outcomes. We cite this literature only to situate and delimit the slower, pre-shot decision regime examined here, not as a theoretical basis for the present hypotheses or for our interpretation of the results. Both remain framed throughout within ecological dynamics.

### 1.4 The present study

The present study examined tee-shot decision-making on dogleg holes under real on-course conditions, explicitly applying this decomposition to separate the two levels of strategic variation. Three hypotheses were proposed:

#### 1.4.1 H1 (exploratory between-player strategy tendency)

We tested the exploratory hypothesis that a player’s tendency toward aggressive or conservative tee-shot engagement, estimated across the three study holes, would be associated with score-to-par. This tendency was indexed by only three decisions and therefore provides a limited index of any stable strategic preference. H1 was accordingly intended to evaluate the usefulness of a coarse player-level strategy measure rather than to provide a definitive test of stable strategic dispositions. Examining this between-player component nonetheless provides a preliminary empirical test of the “stable player-type” assumption common in golf-strategy discussions. Specifically, it asks whether characterising players as broadly aggressive or conservative carries explanatory weight for scoring beyond their situational, within-player engagement with the affordances of each hole.

#### 1.4.2 H2 (within-player situational decision)

Within the same player, situational deviations toward aggressive tee-shot decisions are expected to be associated with higher scores relative to par. Aggressive decisions on dogleg holes often involve attempting to exploit affordances whose execution demands may exceed the precision achievable in a given situation, yielding an expected association with higher score-to-par on average.

#### 1.4.3 H3 (exploratory ability-dependent moderation)

As a secondary exploratory hypothesis, we examined whether the within-player association between aggressive engagement and score-to-par varied across individuals as a function of baseline performance. We expected that weaker baseline performers might show larger within-player decision–performance associations, although this interpretation remained tentative because affordance perception and action capabilities were not directly measured. Given that this hypothesis depends on individual-level variance estimates that are inherently uncertain with only three observations per player, H3 is treated as exploratory throughout.

## 2 Methods

### 2.1 Participants

Eighty-one male university golfers (mean age = 20.79 years, SD = 1.31) participated in this study. Participants were recruited by invitation from a university golf training programme; the inclusion criteria were current enrolment in the programme, the ability to complete the three target holes, and being injury-free at the time of testing. We therefore characterise the sample as amateur, non-elite. The mean handicap was 25.99 (SD = 9.50, range: 8–49). Handicap is the standard golf performance index calibrated so that higher values indicate lower playing ability (a scratch player has handicap 0; recreational amateurs commonly range from 10 to 36). The mean driving distance was 214.50 m (SD = 45.20, range: 120–320 m). Driving distance refers to the recorded distance of full tee shots from the tee; in the present study it was measured as a baseline capability indicator in an independent launch monitor-based session, conducted separately from the three study holes. No launch-monitor or shot-tracking data were collected during play on the three dogleg holes. Launch monitors are devices (typically radar-based, camera-based, or both) that estimate ball-flight parameters and shot distance. Weekly training volume and years of playing experience were not systematically recorded in the present dataset, which we acknowledge as a limitation in describing the sample. All participants provided written informed consent. Study procedures conformed to the ethical standards of the institutional research ethics committee.

### 2.2 Study design and holes

This study employed a field-based observational design. Three dogleg holes were selected on a regulation outdoor golf course (Figure 1). Hole 6 (par 5, left dogleg, stroke index 8) was the longest and easiest-rated of the three. Hole 11 (par 4, left dogleg, stroke index 2) was a high-difficulty par 4. Hole 18 (par 4, right dogleg, stroke index 1) was rated the most difficult hole on the course. The three holes thus varied in par value, difficulty rating, and dogleg direction. All participants played from the blue tee box on each hole. The three holes were selected because they all featured a clearly discernible aggressive-vs-conservative tee-shot choice on the same regulation course, and together they varied in par (5/4/4), stroke index (8/2/1) and dogleg direction (left/left/right). This sampling captures within-course variation in the affordance landscape while keeping the course and teeing context constant and execution-relevant conditions broadly comparable across the three holes. These three holes were, moreover, the only holes on the course that offered clearly comparable dogleg tee-shot affordances under the same teeing context, which is why the design was limited to three decisions per player.

### 2.3 Procedure and measures

Before each tee shot, participants verbally reported their intended strategy (aggressive or conservative). This report was recorded by the researcher before the shot was executed, ensuring that decisions reflected pre-shot planning rather than post-hoc rationalisation. After the strategy report, players completed the hole under standard golf rules, and the total number of strokes was recorded. Score-to-par was calculated as strokes taken minus par and served as the primary outcome variable; positive values indicate scores above par. No systematic shot-tracking or visual coding of actual ball trajectory was performed. The analyses below are based on declared pre-shot intended strategy rather than objectively verified shot trajectories.

Tee-shot decision was coded as 1 (aggressive) or 0 (conservative). We note at the outset that this binary coding is a pragmatic operationalisation of each player’s declared pre-shot orientation toward the affordance landscape, and is not intended to imply that decision-making is a discrete cognitive event separated from action. The coded decision was decomposed following the within-between framework (Curran and Bauer, 2011; Rohrer and Murayama, 2023): the between-player component (decision between) is each player’s mean proportion of aggressive decisions across three holes, representing stable strategic tendency; the within-player component (decision within) is the deviation of each individual decision from that player’s own mean, capturing situational variation. The final analytic sample comprised 81 male university golfers, each contributing one tee-shot observation on each of the three dogleg holes, yielding 243 observations (81 × 3). No trial-level decision or score values were missing in the analytic dataset. To illustrate, a player who chose the aggressive line on two of three holes has a between-player aggressiveness of 0.67; their within-player deviation is +0.33 on the two aggressive holes and −0.67 on the conservative hole. The within-player coefficient therefore quantifies the change in score-to-par associated with situational deviation from each player’s own typical engagement, not with absolute strategy choice.

### 2.4 Statistical analysis

The data have a multilevel structure (three repeated tee-shot observations nested within each player), which calls for a multilevel model so that stable between-player differences and situational within-player decision variation can be estimated separately. We adopted a Bayesian framework because it expresses uncertainty directly through posterior distributions [e.g., 95% credible intervals and *p* (*β* > 0)], and because the robustness of conclusions to prior specification can be quantified through formal prior sensitivity analyses. The priors used below are weakly informative regularisation tools and do not represent any claim about athletes’ static decision-making capabilities; this matters because the ecological dynamics framing of decision-making is, in principle, dynamic and process-based.

All analyses were conducted in R using the brms package (Bürkner, 2017). A Bayesian multilevel regression model (M1) was estimated with score-to-par as the outcome. The model included between-player decision tendency and within-player decision deviation as separate predictors, alongside standardised handicap, standardised driving distance, and hole identity (reference: Hole 11) as fixed effects, with a random intercept for player. Weakly informative priors were specified: Normal (0, 1) for regression coefficients, Student-t (3, 0, 2.5) for the intercept, and Exponential (1) for standard deviation parameters. Posterior distributions were estimated using the No-U-Turn Sampler with four chains of 4,000 iterations each (2,000 warm-up), yielding 8,000 posterior draws. Convergence was confirmed by R̂ = 1.00 for all parameters. While score-to-par on a single hole is discrete and bounded, its empirical distribution in this sample spans roughly −1 to +5 with no severe ceiling/floor effects in the relevant range. We adopted a Gaussian likelihood for the primary model because it (a) yields directly interpretable effects in stroke units (e.g., “half a stroke per hole”) and (b) is widely understood. As a robustness check, we re-estimated the model with a cumulative ordinal likelihood (M4).

Parameter estimates are reported as posterior means with 95% credible intervals (CrI). Effects were interpreted as credible when the 95% CrI excluded zero. The posterior probability that each coefficient was positive, *p* (*β* > 0), was reported to characterise directional evidence. For the between-player effect, a Region of Practical Equivalence (ROPE) analysis was conducted using the interval [−0.10, 0.10] (Kruschke, 2018, 2021). Model fit was evaluated using posterior predictive checks; the Bayesian *R*^2^ (Gelman et al., 2019) and intraclass correlation coefficient (ICC) were computed. Nested models were compared using leave-one-out information criterion differences.

Three interaction models (M2a–M2c) tested whether the within-player decision effect was moderated by hole identity, handicap, and driving distance, respectively. A random slope model (M3) estimated individual variation in the within-player decision effect, with the correlation between random intercepts and slopes examined as exploratory evidence regarding ability-dependent moderation. To assess the robustness of the intercept-slope correlation, prior sensitivity analyses re-estimated M3 under four LKJ priors: LKJ (1), LKJ (2), LKJ (4), and LKJ (10), spanning from uniform to strong shrinkage toward zero. The sensitivity of fixed effects to regression coefficient priors was also examined using narrow [N (0, 0.5)] and wide [N (0, 5)] alternatives.

## 3 Results

### 3.1 Descriptive statistics

Players selected the conservative strategy in 65.0% of tee shots (*n* = 158) and the aggressive strategy in 35.0% (*n* = 85). Strategy selection varied by hole: Hole 11 showed the most balanced distribution (53.1% conservative, 46.9% aggressive), while Hole 6 elicited the highest proportion of conservative decisions (74.1%). Fifty-seven players (70.4%) were classified as predominantly conservative and 24 (29.6%) as predominantly aggressive based on their mean decision tendency (see Table 1).

**Table 1:** Descriptive statistics for participant characteristics, decision frequencies, and decision style classification.

Variable	n/N	M/%
Participant characteristics		
Age (years)	81	20.79
Handicap (HDCP)	81	25.99
Driving distance (m)	81	214.50
Decision frequency		
Overall conservative	158	65.0%
Overall aggressive	85	35.0%
Hole 11 conservative	43	53.1%
Hole 11 aggressive	38	46.9%
Hole 18 conservative	55	67.9%
Hole 18 aggressive	26	32.1%
Hole 6 conservative	60	74.1%
Hole 6 aggressive	21	25.9%
Decision style classification		
Conservative players	57	70.4%
Aggressive players	24	29.6%

Decision style was classified based on the player-level mean proportion of aggressive decisions: players with a mean > 0.50 were classified as aggressive. Percentages for decision frequency are calculated within each hole. All 243 observations from 81 players (81 × 3) are included in the descriptive statistics.

The mean score-to-par across all 243 observations was approximately +2.0 strokes. Hole 11 produced the highest mean score-to-par (*M* = 2.28, SD = 1.49), followed by Hole 18 (*M* = 2.06, SD = 1.20) and Hole 6 (*M* = 1.64, SD = 1.00). Descriptively, conservative decisions were associated with lower mean score-to-par (*M* = 1.87, SD = 1.05) than aggressive decisions (*M* = 2.24, SD = 1.59), consistent with the direction of the within-player model effect, though this comparison does not account for ability or data structure. The higher mean score-to-par on Hole 11 and lower mean on Hole 6 are reflected in the fixed-effect estimates reported in Section 3.2 (Hole 6 credibly lower than Hole 11; Hole 18 not credibly different). Classifying players as predominantly conservative or aggressive based on three decisions is used here as a descriptive convenience and does not warrant inferences about stable player types; the inconclusive between-player effect in Section 3.2.1 confirms that the current design does not support such inferences.

### 3.2 Bayesian multilevel model

The model converged satisfactorily (all R̂ = 1.00; bulk ESS > 1,400; tail ESS > 2,000 for all parameters). Posterior predictive checks indicated adequate capture of central tendency and variability, though the Gaussian likelihood did not fully reproduce the discrete, multimodal character of the observed distribution. The Bayesian *R*^2^ was 0.29 [95% CrI (0.16, 0.40)], indicating that a substantial proportion of variance in score-to-par remained unexplained; additional variables such as shot accuracy, approach lie quality, and within-round psychological state are likely to account for this residual variance. The ICC was 0.17, indicating that approximately 17% of total variance was attributable to stable between-player differences. Posterior estimates for all parameters are reported in Table 2.

**Table 2:** Posterior estimates from the Bayesian multilevel regression model predicting score-to-par.

Parameter	Estimate	Est.error	l-95% CrI	u-95% CrI	*p* (*β* > 0)
Fixed effects					
Intercept	2.12	0.16	1.80	2.44	> 0.999*
Decision (between)	0.26	0.28	−0.29	0.80	0.818
Decision (within)	0.51	0.20	0.11	0.90	0.993*
Handicap (z)	0.37	0.13	0.13	0.63	0.998*
Driving distance (z)	0.04	0.13	−0.20	0.29	0.624
Hole 18	−0.14	0.17	−0.48	0.20	0.210
Hole 6	−0.52	0.17	−0.85	−0.18	0.001*

CrI, credible interval. *p* (*β* > 0), posterior probability that the parameter is positive. * indicates that the 95% CrI excludes zero. Hole 11 serves as the reference category.

#### 3.2.1 Between-player decision tendency

**Figure 2:**
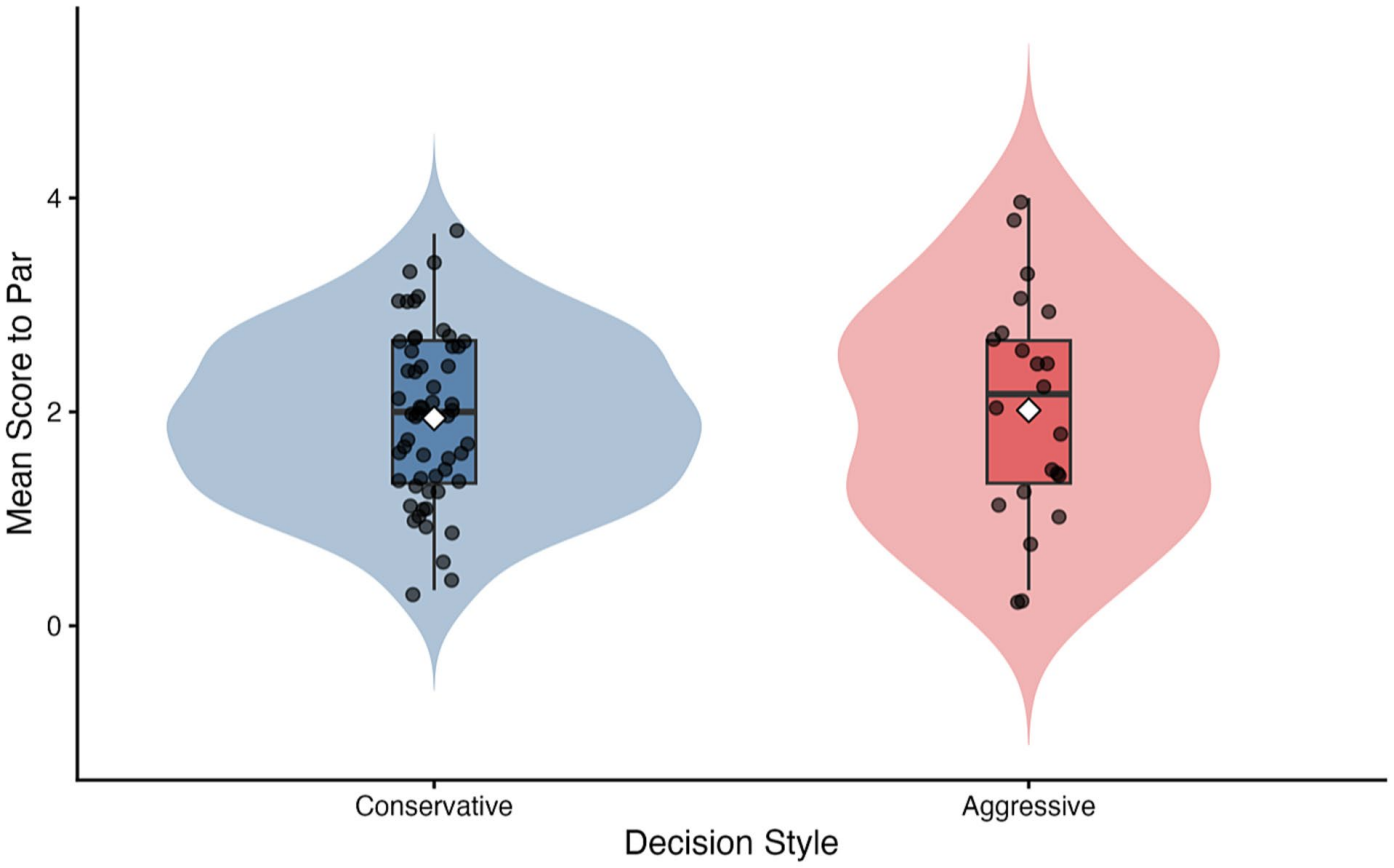
Distribution of mean score-to-par by decision style classification. Players were classified as conservative (*n* = 57) or aggressive (*n* = 24) based on whether their mean proportion of aggressive decisions exceeded 0.50. The two groups exhibited broadly overlapping distributions, consistent with the inconclusive between-player effect in the multilevel model.

The between-player effect was small and uncertain [Estimate = 0.26, 95% CrI (−0.29, 0.80), *p* (*β* > 0) = 0.818]. The result is inconclusive: the posterior does not provide clear evidence of a systematic difference in score-to-par between players who generally favoured aggressive versus conservative strategies, but it equally cannot support a conclusion of no effect. A ROPE analysis revealed that only 18.7% of the posterior distribution fell within the region of practical equivalence [−0.10, 0.10]. The overlap between the two descriptive groups (Figure 2) is consistent with this inconclusive between-player effect.

#### 3.2.2 Within-player decision choice

The within-player effect was positive and credible [Estimate = 0.51, 95% CrI (0.11, 0.90), *p* (*β* > 0) = 0.993]. When the same player adopted a more aggressive strategy on a particular hole relative to their typical tendency, score-to-par tended to be approximately half a stroke higher. Situational deviations toward aggressive decisions were thus associated with poorer hole performance.

#### 3.2.3 Player ability and hole effects

Handicap was positively associated with score-to-par [Estimate = 0.37, 95% CrI (0.13, 0.63), *p* (*β* > 0) = 0.998], confirming that higher-handicap players recorded higher scores relative to par. Driving distance showed no credible association [Estimate = 0.04, 95% CrI (−0.20, 0.29)], indicating that the decision-performance relationship was not attributable to differences in driving distance. Hole 6 was associated with lower score-to-par relative to Hole 11 [Estimate = −0.52, 95% CrI (−0.85, −0.18)]; Hole 18 did not differ credibly from the reference.

#### 3.2.4 Interaction effects and random slope model

No clear evidence for moderation was found in the predefined interaction models: the interactions of within-player decision with hole identity (Δelpd = −1.7, SE = 0.8), handicap [Estimate = −0.04, 95% CrI (−0.43, 0.35)], and driving distance [Estimate = −0.04, 95% CrI (−0.42, 0.33)] were all negligible and not credible.

**Figure 3:**
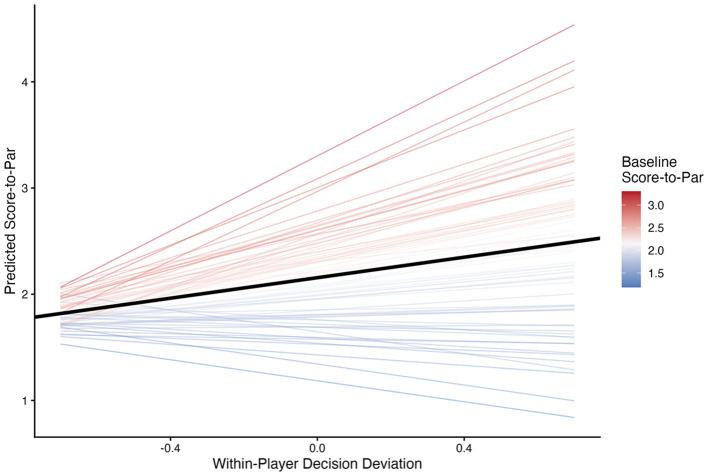
Individual variation in the decision-performance relationship, estimated from the random slope model. Each line represents one player’s estimated slope linking within-player decision deviation to predicted score-to-par; line colour reflects baseline score-to-par (blue: stronger performance; red: weaker performance). The fan-shaped pattern, in which red lines tend to be steeper, illustrates the positive intercept-slope correlation [*r* = 0.70, 95% CrI (0.14, 0.96)]. Prior sensitivity analyses indicated that this correlation was sensitive to prior specification and should be interpreted with caution.

The random slope model (M3) revealed substantial individual heterogeneity in the within-player decision effect [slope SD = 0.79, 95% CrI (0.23, 1.29); see Figure 3 for individual slopes]. The population-level within-player effect remained credible in this specification [Estimate = 0.48, 95% CrI (0.05, 0.91)]. The correlation between random intercepts and slopes was positive [*r* = 0.70, 95% CrI (0.14, 0.96)], providing exploratory evidence that players with higher baseline scores-to-par may tend to show larger within-player associations between aggressive engagement and score-to-par. Note that this correlation was sensitive to prior specification; see prior sensitivity analyses in the Results section.

Prior sensitivity analyses examined the robustness of this intercept-slope correlation. Under LKJ priors ranging from LKJ (1) to LKJ (10), the posterior mean of r decreased from 0.83 to 0.25. The 95% CrI included zero under LKJ (4) and LKJ (10), indicating that the credibility of the correlation is sensitive to prior specification. The direction remained consistently positive across all priors [*p* (r > 0) ≥ 0.877]. In contrast, fixed-effect estimates were substantively unchanged under both narrow [N (0, 0.5)] and wide [N (0, 5)] regression coefficient priors relative to the original prior, confirming that the main-effects results are robust to prior specification.

A cumulative ordinal model (M4) was estimated to assess whether the within-player decision effect was robust to the Gaussian distributional assumption. The ordinal model yielded a positive and credible within-player decision effect [Estimate = 0.87, 95% CrI (0.15, 1.58), *p* (*β* > 0) = 0.991, on the log-odds scale], consistent in direction and credibility with the primary Gaussian model. This indicates that the association between within-player aggressive decisions and higher scores is not an artefact of the distributional assumption.

## 4 Discussion

This study examined tee-shot decision-making on dogleg holes in relation to hole performance. Three findings emerged. First, the present data did not provide reliable evidence for an effect of players’ stable strategic tendencies on score-to-par, though this result should be interpreted cautiously given the limited measurement basis. Second, situational deviations toward aggressive decisions within players were credibly associated with higher scores, amounting to approximately half a stroke per hole. Third, the random slope model provided exploratory evidence that this within-player association may be larger for players with weaker baseline performance, though the intercept-slope correlation underlying this inference was sensitive to prior specification. Together, these findings are compatible with an ecological dynamics interpretation in which players’ situated engagement with dogleg affordances, constrained by task goals and action capabilities, is associated with scoring outcomes. Because affordance perception and action calibration were not directly measured, this interpretation remains inferential rather than a mechanism tested by the present design.

### 4.1 Stable strategic tendencies and performance

The between-player effect of strategic tendency was small and uncertain, with the posterior reflecting neither a credible positive effect nor practical equivalence to zero. This result should not be interpreted as evidence that stable strategic tendencies are unimportant. Each player’s tendency was estimated from only three decisions, a sample too small to yield a reliable estimate of a stable preference. The null result for H1 may therefore reflect measurement limitations rather than the genuine absence of an effect, and future research using larger numbers of holes per player would be needed to adequately test the role of stable tendencies.

From an ecological dynamics perspective, the absence of a credible between-player effect is theoretically interpretable. Because the affordance structure of each dogleg hole varies, a fixed aggressive or conservative preference would be expected to prove advantageous in some contexts but disadvantageous in others, yielding no reliable net benefit across situations (Seifert et al., 2021, 2024). The present data are consistent with this reasoning, as outlined in the Introduction, though they do not provide direct evidence for the underlying mechanism. Importantly, the present data cannot distinguish between a genuinely negligible between-player effect and between-player effects that cancel out across heterogeneous hole contexts; the ecological dynamics interpretation should therefore be read as one account compatible with the observed pattern, not as an account that the present design has tested.

### 4.2 Situational decisions and scoring associations

The within-player effect of aggressive decisions was positive and credible. A deviation toward an aggressive strategy on a particular hole, relative to a player’s own typical tendency, was associated with a higher score-to-par of approximately half a stroke. While modest in isolation, this effect is practically meaningful given the cumulative nature of golf scoring: across multiple dogleg holes in a round, repeated aggressive selections followed by higher scores could accumulate to one or more strokes, a margin that frequently separates finishing positions in on-course play. The posterior probability of 99.3% provides strong directional evidence. This finding complements the strokes-gained framework (Broadie, 2014; Broadie and Hurley, 2017) by showing that declared pre-shot strategic choices are associated with scoring variance, in addition to the execution-related factors emphasised in that literature.

Within the ecological dynamics framework, this pattern may reflect instances of misalignment between perceived affordances and effective action capabilities: a player perceives the aggressive route as viable, but the execution demands of that route exceed what the player can reliably produce in that specific situation (Kiverstein et al., 2021; Seifert et al., 2021). It should be noted that affordance perception was not directly measured; this interpretation is offered as one account consistent with the data rather than a tested mechanism.

An important interpretive boundary should be acknowledged. The present design cannot distinguish between strategy quality and execution quality. Score-to-par reflects the combined result of the decision made before the shot and the quality of shot execution. It is possible that an aggressive line is the objectively correct choice for a given player on a given hole but execution error yields a poor outcome, or conversely that a well-executed aggressive shot yields a favourable result. For this reason, aggressive decisions are described throughout as being associated with poorer outcomes rather than as inherently poor choices. Put directly, the present study estimates the net outcome associated with declared strategy rather than the isolated causal effect of decision quality. Future research incorporating intermediate shot outcome variables, such as landing position or approach distance, would allow the separate contributions of decision and execution to be estimated (Broadie, 2012, 2023).

The possibility of reverse causality also warrants acknowledgement. Players performing poorly during a round may adopt more aggressive strategies on subsequent holes as compensatory risk-taking. Several features of the design mitigate this concern: decisions were reported before each shot rather than retrospectively; the within-between decomposition controls for each player’s baseline tendency; and the three study holes were played as non-consecutive tasks on the course, with other holes played between them, reducing the likelihood of direct sequential carry-over. Nevertheless, future within-round sequential designs would be needed to more definitively address the direction of the decision-performance relationship.

### 4.3 Individual heterogeneity and prior sensitivity

The predefined interaction tests found no credible evidence that the decision-performance relationship was moderated by handicap or driving distance. This result should be distinguished from the random slope finding, which addresses a different question. Whereas fixed-effect interactions test whether specific measured variables linearly moderate the decision effect, the random slope model captures total individual variation in that effect regardless of its source. The positive intercept-slope correlation (*r* = 0.70) suggests that this heterogeneity may be related to overall baseline performance, but prior sensitivity analyses demonstrated that this correlation is sensitive to prior specification: the 95% CrI included zero under LKJ (4) and LKJ (10), even as the direction remained consistently positive. The H3 finding should therefore be regarded as a preliminary exploratory observation rather than a confirmed result.

Any interpretation of this exploratory pattern should remain tentative. If the positive intercept-slope correlation were replicated in larger designs, the data would be consistent with the possibility that players with weaker baseline performance show larger within-player associations between aggressive engagement and score-to-par, reflecting wider gaps between the perceptual attunement and action capabilities required to realise the corner-cut affordance. Testing such an account would require direct measurement of perceptual attunement and action capabilities, which the present study did not include. The sensitivity of the intercept-slope correlation to prior specification, combined with the limited number of observations per player, underscores the need for caution in drawing conclusions from this exploratory finding.

### 4.4 Applied demonstration of within-between decomposition in field-based golf decision research

The within-between decomposition applied here is an established multilevel modelling technique (Curran and Bauer, 2011; Rohrer and Murayama, 2023); the contribution of the present study is applied rather than methodological. Specifically, we demonstrate (a) that the technique can be used productively in a real on-course golf setting; (b) that pre-shot verbal reports provide a feasible way to capture declared strategy without resorting to post-hoc rationalisation; (c) that the two levels of variation (between-player tendency and within-player situational deviation) can yield qualitatively different conclusions even within the same sample; and (d) that the approach can be applied to other sport contexts in which repeated strategic choices are made under varying environmental constraints (Gleeson and Kelly, 2020; Raab and Johnson, 2004). The collection of decision data under real playing conditions, with intentions recorded before rather than after the shot, further strengthens the ecological validity of the findings (Broadie, 2012). The within-between decomposition demonstrated here may also be informative for other slower-paced strategic contexts in sport in which repeated decisions are made under varying environmental constraints.

### 4.5 Practical implications

The present results suggest that declared pre-shot strategy is associated with scoring variance even after accounting for handicap and driving distance, although the present design cannot isolate strategy quality from shot execution quality (Lauriola et al., 2014; Nagengast et al., 2011). Rather than prescribing a fixed strategy across players or contexts, coaching could foster perceptual attunement to the ecological information specifying the corner-cut affordance, such as the visible width of the fairway, the position of non-fairway penalty areas, wind direction, tee-box angle, and the player’s current ball-flight tendencies. This perceptual attunement should develop alongside the action capabilities required to realise that affordance reliably. From a constraints-led perspective, coaches might support this process by manipulating practice constraints on dogleg holes: for example, asking players to attempt the same hole from different tee positions, using markers to vary the apparent width of safe landing zones, or requiring different clubs to alter the carry distance needed to realise the corner-cut affordance. Coaches might also vary the apparent proximity of hazards along the corner, or have players rehearse the same hole under different wind conditions, in each case holding the task goal constant so as to progressively refine players’ attunement to when the aggressive line is actionable. Players could also compare their intended line with the actual landing position after each shot, using this feedback to recalibrate their perception of whether the aggressive route is currently actionable. If replicated in larger designs, the exploratory pattern of larger scoring differences associated with declared aggressive strategy among higher-handicap players (Gnagy et al., 2015) may inform developmentally graded course-management guidance, though this recommendation should be treated with caution pending replication.

### 4.6 Limitations and future directions

Several limitations should be acknowledged. First, only three dogleg holes from a single course were examined. Future research could examine a more diverse set of holes across multiple courses, incorporating structural features such as fairway width, corner severity, and hazard placement as moderators. Second, strategic decisions were measured using a binary self-report classification, which simplifies a likely continuous spectrum of strategic options and may not fully capture decision processes. Because actual tee-shot trajectories were not coded, intended-aggressive shots that were mishit into conservative landing areas, or intended-conservative shots that deviated toward aggressive lines, could not be separated; this limitation may be especially relevant for higher-handicap players. Future shot-tracking technology could provide more fine-grained and objective measures of intended and actual strategy. As noted in the Methods, and consistent with an ecological dynamics perspective, this binary verbal classification is a pragmatic observational coding rather than evidence that decision-making is a discrete cognitive event separated from action (Carvalho and Araújo, 2022). Third, the between-player tendency was estimated from only three decisions per player, limiting the reliability of that construct and the interpretability of H1. Fourth, the Gaussian likelihood did not fully reproduce the discrete character of score-to-par. A robustness check using a cumulative ordinal model confirmed that the within-player decision effect was consistent in direction and credibility with the primary Gaussian model, suggesting that the main conclusions are not dependent on the distributional assumption. Fifth, the sample consisted exclusively of male university golfers, limiting generalisation to other populations. Finally, the cross-sectional observational design precludes causal inference: the observed association may reflect reverse causality, unobserved confounding, or both.

## 5 Conclusion

This study provides field-based evidence that tee-shot strategic decision-making is associated with scoring outcomes on dogleg holes. The within-player effect of aggressive decisions was credible and robust to alternative regression-coefficient priors and to an ordinal-likelihood robustness check, amounting to approximately half a stroke per hole. The between-player effect of stable strategic tendency was inconclusive, a result attributable at least in part to the limited number of decisions per player. Exploratory evidence from the random slope model suggested that the within-player association between aggressive engagement and score-to-par may be larger for players with weaker baseline performance, though this finding was sensitive to prior specification and should be treated as preliminary. These conclusions should be interpreted as observational associations from a male university amateur sample playing three dogleg holes on a single regulation course, rather than as causal evidence that aggressive decisions necessarily worsen performance. Together, these results highlight the value of applying within-between decomposition to sport strategy research and support the view that course management training represents a meaningful complement to technical skill development in golf.

## Data Availability

The raw data supporting the conclusions of this article will be made available by the authors, without undue reservation.
